# Supplementary material for: DNA origami signal amplification in lateral flow immunoassays
Source: Nat Commun. 2025 Apr 4;16:3216. doi: 10.1038/s41467-025-57385-6 (PMC11971289; doi:10.1038/s41467-025-57385-6)
Supplement: Supplementary file 1 — Supplementary Information [file 41467_2025_57385_MOESM1_ESM.pdf]

Supporting Information for
DNA Origami Signal Amplification in Lateral Flow Immunoassays

Heini Ijäs *, Julian Trommler, Linh Nguyen, Stefan van Rest, Philipp C. Nickels, Tim Liedl, Maximilian J. Urban *

Faculty of Physics and Center for NanoScience (CeNS), Ludwig-Maximilians-University, Geschwister-Scholl-Platz 1, Munich, 80539, Germany.

Contents

Page

Supplementary Methods **3**
Neurofilament light chain (NfL) LFIAs 3
Commercial cTnI assays 3

Supplementary Notes **4**
Note 1: Supplementary data of antibody-DNA conjugation (Fig. S1) 4
Note 2: AGE analysis of antibody-6HB conjugate formation (Fig. S2) 5
Note 3: Additional TEM images of antibody-6HB conjugates (Fig. S3–S4) 6
Note 4: Additional TEM images of 6HBs with 40 nm gold-DNA labels (Fig. S5) 8
Note 5: AGE analysis of the attachment of A647-DNA labels on 6HBs (Fig. S6) 9
Note 6: Fluorescence strip images with 0 nmol/L biotin-DNA in water, serum, and saliva (Fig. S7) 10
Note 7: Full LFIA test strip images from cTnI fluorescence assays (Fig. S8) 11
Note 8: NfL detection in human serum (Fig. S9) 12
Note 9: Screening of antibody pairings for cTnI detection with the A647 fluorescence assay (Fig. S10) 14
Note 10: Comparison of the sensitivity of DNA origami signal amplification to commercial cTnI lateral flow assays (Fig. S11) 15
Note 11: Supplementary data for cTnI LoD determination (Fig. S12–S13) 17
Note 12: The 6HB design (Fig. S14, Table S1) 19
Note 13: cTnI LFIAs with different antibody-origami ratios (Fig. S15) 24

Unprocessed Gel Scans **25**

Supplementary References **29**

Supplementary Methods

Neurofilament light chain (NfL) LFIAs

Antibodies for neurofilament light chain (NfL) sandwich immunoassays were purchased from Uman Diagnostics (Umeå, Sweden). Anti-NfL antibody UD1 was used as the detection antibody and biotinylated anti-NfL UD3 as the capture antibody. The UD1 antibody was covalently labeled with the 21-nt DNA oligonucleotide handle according to the protocol described in the main text for the anti-cTnI antibodies, with the exception that a 5-fold molar excess of the DBCO-PEG$_5$-NHS crosslinker was used in the first conjugation step instead of a 15-fold excess. The DNA-labeled antibodies were purified using ion exchange chromatography (Dynamic Biosensors), in which the DNA-labeled antibodies were eluted over a salt gradient from 100% buffer A (25 mmol/L Na_2HPO_4/NaH_2PO_4, 150 mM NaCl, pH 7.2) to 25% of buffer A and 75% buffer B (25 mmol/L Na_2HPO_4/NaH_2PO_4, 1 M NaCl, pH 7.2) over the course of 16 minutes at a flow rate of 1 mL/min. The collected fractions with DNA-labeled antibodies were upconcentrated using 100k Amicon Ultra centrifugal filters (Merck Millipore). Antibody-DNA origami conjugates were prepared by mixing 6HB DNA origami structures with a 3-fold molar excess of the purified antibody-DNA conjugates and with a 100-fold molar excess of A647-DNA labels and incubating the mixtures at +4 °C for a minimum of 2 hours before use.

The NfL LFIAs were run on Milenia GenLine HybriDerect Variant lateral flow test strips with streptavidin and anti-dig test lines and an anti-goat control line. For LFIAs with gold-DNA, the labels were dried on the sample application pad of the test strips as described in the main text. 1:5 dilutions of recombinant human 68 kDa neurofilament/NF-L protein (His tag) (Abcam) from 156.25 nmol/L to 2 pmol/L were prepared in human serum (Sigma-Aldrich). LFIAs with A647-DNA labels were then run according to the LFIA running protocol described in the main text for cTnI, with the exception that the concentration of 6HB was 8 nmol/L 6HB, and the concentration of the biotinylated capture antibody was 60 nmol/L instead of 120 nmol/L before mixing with the sample. An unamplified reference assay was prepared with the same anti-NfL antibody combination at an identical antibody concentration as in the 6HB sample, corresponding to 8 nmol/L of the DNA-labeled detection antibody, 267 nmol/L of A647-labeled oligonucleotide for hybridizing with the DNA handles on the detection antibody, and 6.67 nmol/L of biotinylated capture antibody, after mixing both antibodies with 5 μL of sample. The tests were imaged and image analysis was carried out as described in the main text.

Commercial cTnI assays

Commercial cTnI lateral flow tests from two different manufacturers were obtained to compare DNA origami signal amplification and in-house produced antibody-gold conjugates to state-of-the-art assays. ACCU-TELL Troponin I Cassettes for whole blood, serum, and plasma (referred to as "commercial test A") were purchased from AccuBioTech Co., Ltd. (Beijing , P. R. China). All-Test Cardiac Troponin I Rapid Test Cassettes for whole blood, serum, and plasma (referred to as "commercial test B") were purchased from Hangzhou AllTest Biotech Co., Ltd. (Hangzhou, P. R. China).

Spiked serum samples for the experiments were produced by preparing dilutions of cTnIC (HyTest Ltd.) in human serum (Sigma-Aldrich) at 6.25 nmol/L, 1.25 nmol/L, 250 pmol/L, 50 pmol/L, 10 pmol/L, 2 pmol/L, 400 fmol/L, and 80 fmol/L concentrations. Commercial test A strips were run with either 50 μL or 5 μL of serum samples, where 50 µL corresponds to the volume recommended by the manufacturer, and 5 μL corresponds to the volume used on the DNA origami test strips. With 50 μL of sample, the sample was first transferred into the sample well and followed by 40 μL of the running buffer supplied with the test. With 5 μL of sample, the sample was first mixed with 85 μL of running buffer and the mixture was applied to the sample well. Commercial strips B were run with either 75 μL of sample, in which case the sample was applied into the sample well without running buffer, or with 5 μL of sample, in which case the sample was first mixed with 70 μL of the supplied running buffer and the mixture was applied into the sample well. In all cases, the test strips were imaged at 10 minutes after the addition of liquid.

Supplementary Notes

Note 1: Supplementary data of antibody-DNA conjugation

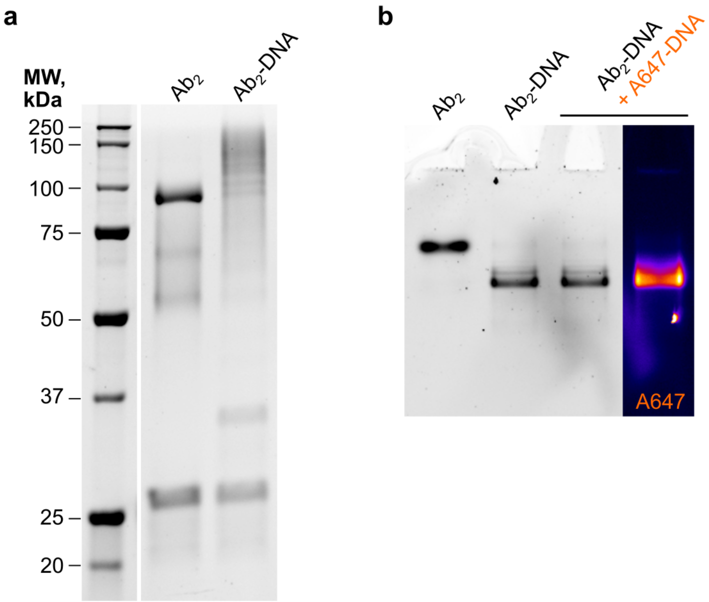

Fig. S1 Gel analysis of antibody-DNA conjugation. **a**. SDS-PAGE analysis of the outcome of labeling Ab_2 (anti-cTnI 19C7cc) with DNA. Left lane (Ab_2) shows unmodified antibody. Ab_2-DNA is the labeling product with a degree of labeling (DoL = n(DNA):n(Ab)) of 2.85. Under denaturing conditions, the IgG heavy chains and light chains carrying one or more conjugated DNA oligonucleotides migrate slower than unmodified protein chains due to the increased molecular weight. **b**. Native PAGE analysis of the DNA labeling of Ab_2. The first two lanes contain the same samples as Fig. S1a. In the third lane, the DNA-labeled antibody was mixed with a 10-fold molar excess of the directly complementary A647-DNA. The non-denaturing conditions of the native PAGE preserve both the quaternary structure of the IgG antibodies as well as DNA hybridization. Under native conditions, the higher negative charge of DNA-labeled antibodies leads to a higher electrophoretic mobility. The binding of the A647-DNA to the DNA handles of the antibody in the third lane can be observed through colocalization of the protein stain signal (left) and the A647 fluorescence (right) after imaging the gel in both channels. Unprocessed gel scans are presented under section "Unprocessed Gel Scans" at the end of the Supplementary Information.

Note 2: AGE analysis of antibody-6HB conjugate formation

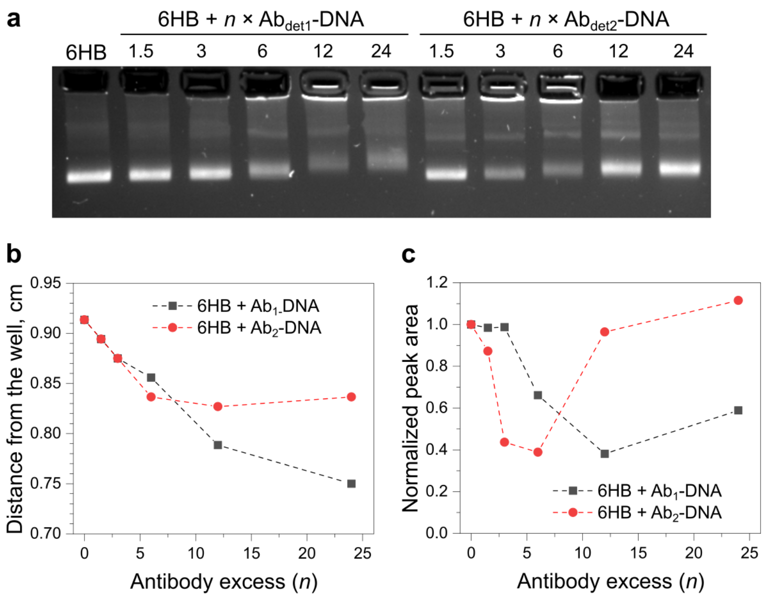

Fig. S2 Characterization of the formation of Ab-6HB conjugates with the detection antibodies Ab_1 and Ab_2. **a**. AGE analysis of Ab-6HB mixtures prepared with different Ab:6HB molar ratios. The reference 6HB sample (leftmost lane) was prepared without antibodies in the same buffer conditions as the Ab-6HB samples. Ab_1-DNA is a DNA-conjugated rabbit anti-cTnI antibody Y302 with a degree of labeling (DoL $= n(\mathrm{DNA}){:}n(\mathrm{Ab})$) of 0.94 determined from the UV-Vis absorbance spectrum of the purified Ab-DNA conjugates. Ab_2-DNA is a DNA-conjugated mouse anti-cTnI antibody 19C7cc with a DoL of 2.85. The unprocessed gel scan is presented under section "Unprocessed Gel Scans". **b**. The electrophoretic mobility of the leading band of samples in Fig. S2a. **c**. The intensity of the leading band in the samples in Fig. S2a.

Note 3: Additional TEM images of antibody-6HB conjugates

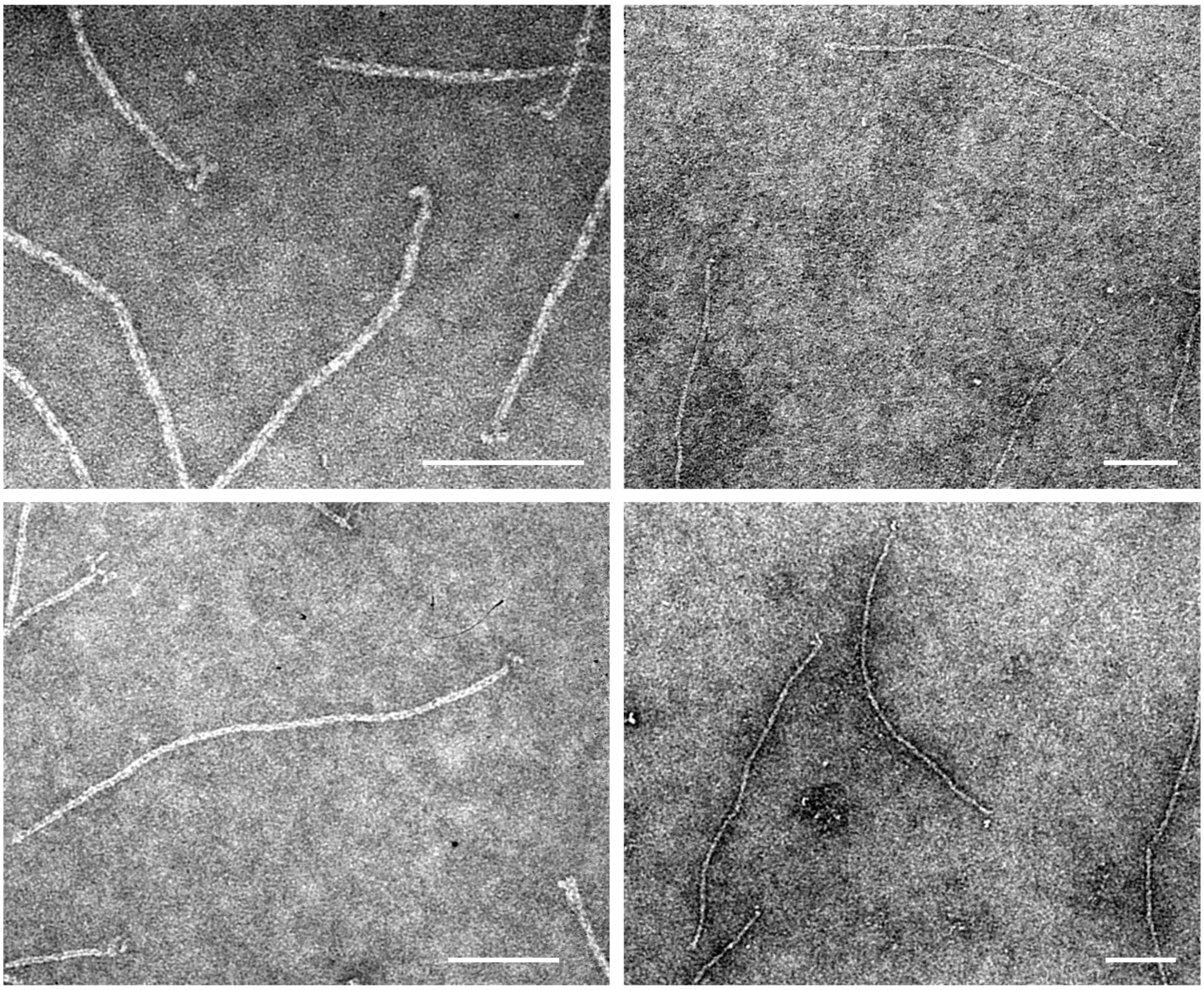

Fig. S3 TEM images of Ab_1-6HB conjugates after incubation of 6HBs with a 6-fold excess of Ab_1-DNA and subsequent gel purification. All scale bars: 100 nm.

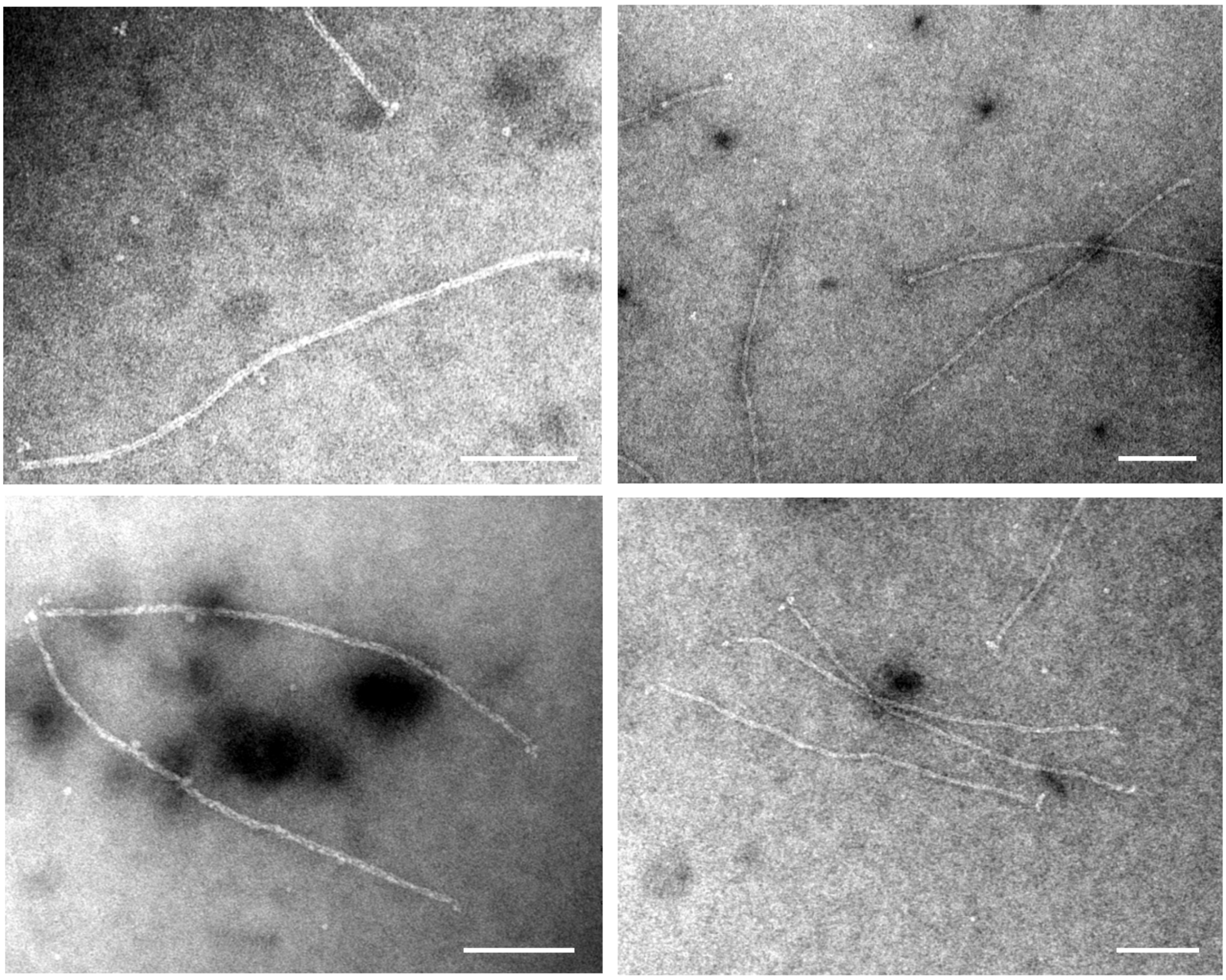

Fig. S4 TEM images of Ab_1-6HB conjugates after incubation of 6HBs with a 6-fold excess of Ab_{det1}-DNA without gel purification. Note that a fraction of antibodies can contain two or more DNA oligonucleotide labels and lead to occasional coupling of two 6HBs, as visualized in the bottom left panel. All scale bars: 100 nm.

Note 4: Additional TEM images of 6HBs with 40 nm gold-DNA labels

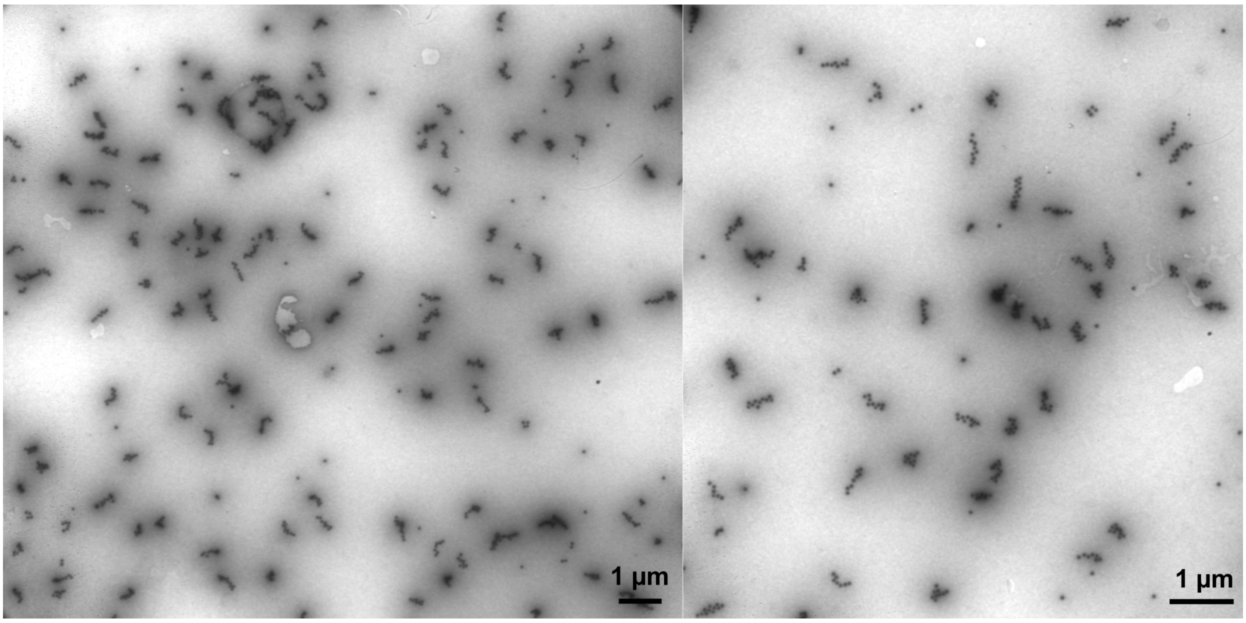

Fig. S5 TEM images of gel-purified 6HBs with 40 nm gold-DNA labels.

Note 5: AGE analysis of the attachment of A647-DNA labels on 6HBs

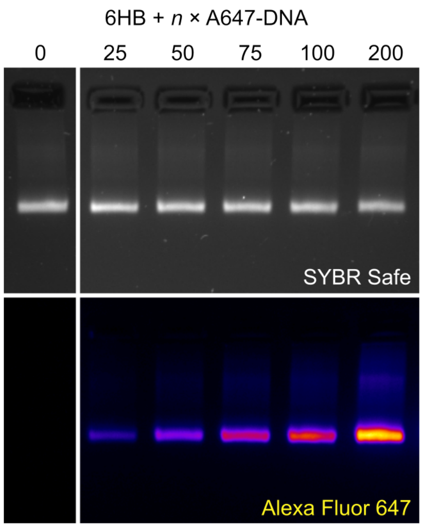

Fig. S6 AGE characterization of mixtures of 6HBs and A647-DNA labels with different A647-DNA:6HB molar ratios. The fluorescence of the SYBR Safe DNA stain is shown in the top panel and the fluorescence of A647 in the bottom panel. The mixtures of A647-DNA labels and 6HBs were incubated at RT for *ca.* 20 minutes before the gel analysis. Unprocessed gel scans are presented under section "Unprocessed Gel Scans"

Note 6: Fluorescence strip images with 0 nmol/L biotin-DNA in water, serum, and saliva

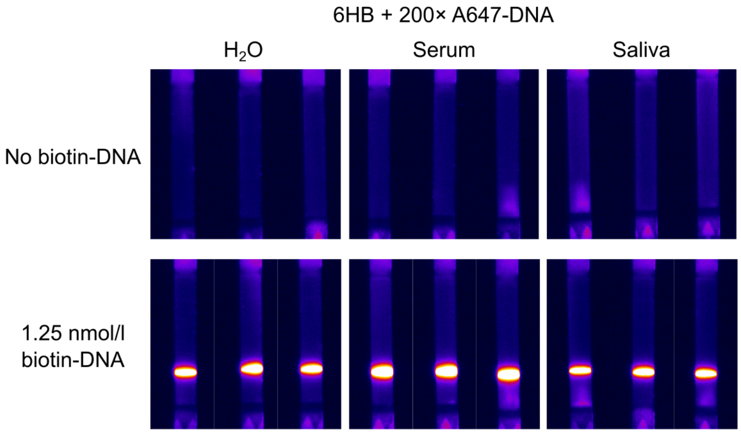

Fig. S7 Fluorescence images of biotin-DNA detection on A647 assay strips with different sample matrices. **The top row** shows blank strips without biotin-DNA analyte; 5 μL of either milli-Q H_2O, human serum, or human saliva was mixed with the 6HBs, applied on the test strip after a short (ca. 1 min) incubation, and flushed over the nitrocellulose membrane with running buffer. The strips were imaged after 15 min running time. For each sample matrix, three repeats are shown. The presented assay strips were run with 6HBs mixed with 200-fold excess of A647-DNA labels before the assay for maximizing potential nonspecific signal. As a reference of the test line intensity in positive samples, **the bottom row** shows three repeats of test strips with 1.25 nmol/L biotin-DNA in each of the tested sample matrices using the same mixture of 6HBs with a 200-fold excess of A647-DNA. The direction of the lateral flow in the strip images is upwards. Due to detection antibodies not being used in the biotin-DNA detection, the anti-rabbit control line on the test strips is not visible.

Note 7: Full LFIA test strip images from cTnI fluorescence assays

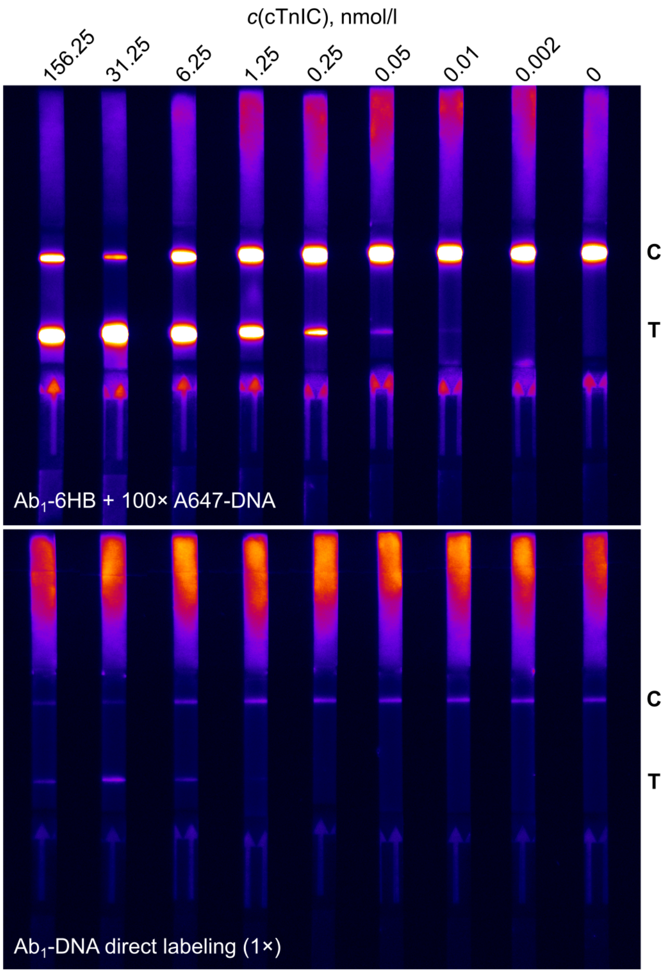

Fig. S8 Fluorescence images of the full cTnI A647-DNA assay test strips for Ab_1-6HB with 100-fold excess of A647-DNA and Ab_1-DNA labeled with a complementary A647-DNA oligonucleotide. All images were captured using a 40 ms exposure time and show the assay result after 15 min run time, thus corresponding to the intensity vs. concentration graph of Fig. 4a in the main text. The locations of the control (C, anti-rabbit) and test (T, streptavidin) lines on the membrane are indicated. The direction of the lateral flow in the images is upwards. For the concentration range of 0–1.25 nmol/L, the test line intensities follow a sigmoidal trend and control line intensities are constant. At high cTnI concentrations (at 6.25 nmol/L cTnI and higher), a considerable fraction of the DNA origami added on the strip (4 nmol/L) are bound to cTnI and captured at the test line, thus reducing the amount of origami at the control line. The so-called hook effect emerges in our experiment at 156.25 nmol/l cTnI; excess cTnI molecules are able to saturate both the detection antibodies on the 6HB and the capture antibodies on the test line, thus preventing sandwich formation at the test line. As a result, the intensity at the test line decreases. Consequently, more 6HBs are carried over the test line and captured at the control line, whose intensity is recovered.

Note 8: NfL detection in human serum

In addition to the cTnI detection described in detail in the main text, the DNA origami signal amplification method can be adapted to the detection of various other analytes. As a second example of applying antibody-6HB conjugates on a sandwich LFIA, we here demonstrate the detection of human NfL in serum samples.

NfL belongs to the group of neuronal cytoskeletal proteins and it has emerged as a clinically relevant biomarker for both acute neuro-axonal injuries and neurodegenerative diseases. [1, 2] NfL can be detected both in the cerebrospinal fluid (CSF) and in the blood. Due to the low concentration of NfL in blood – typically 50–100 times lower than in the CSF – NfL detection has still sensitivity limitations and ultrasensitive methods, such as Simoa, are typically used for blood testing. [3, 4] The literature on NfL detection with simple and cost-effective methods such as lateral-flow based assays is limited. Recently, Zhang *et al.* reported NfL detection at 117.65 pmol/L sensitivity in a lateral flow assay using gold nanoshells, which provide an enhanced sensitivity compared to colloidal gold labels. [5]

To demonstrate NfL detection with DNA origami, we applied the same 6HB signal amplification structure as for the cTnI detection, but combined it with an anti-NfL antibody pair (DNA-labeled detection antibody and a biotinylated capture antibody). NfL was then detected in the A647 assay (Fig. S9). The visual LoD of an unamplified reference assay with the same antibody combination was at 6.25 nmol/L. A visual LoD of 10 pmol/L, corresponding to 680 pg/mL, was reached with antibody-6HB conjugates labeled with a 100-fold molar excess of A647-DNA labels. With this sensitivity, we thus outperform NfL detection with nanoshell-assisted LFIAs 10-fold [5]. For testing of CSF, we are able to cover the full spectrum of levels for healthy (5—30 pmol/L) and diseased individuals (> 30 pmol/L; note, however, that these are rough values that are also strongly age dependent) in dementia and stroke. In the long term, we aim to improve sensitivity further and address clinical unmet needs in the field of rapid blood tests for neurodegenerative diseases and stroke. While our current sensitivity is still one order of magnitude too high for detecting elevated levels in dementia patients [4] and in all stroke patients, it reaches the NfL levels in blood plasma of patients with severe neuro-axonal injuries [6, 7].

The similar sensitivity of the NfL detection to the cTnI assays shown in the main text demonstrates that the DNA origami signal amplification method can be adapted to various analytes and LFIAs through a straightforward process of antibody selection. Here, we chose to follow the sample preparation and assay protocols established for the cTnI assay as closely as possible, with the exception of adjusting the relative concentrations of assay components and using a higher purity of the DNA-labeled detection antibody. At the highest NfL concentrations, particularly visible from 6.25 nmol/L upwards, some extent of DNA origami crosslinking and aggregation in the beginning of the nitrocellulose membrane was observed. This NfL concentration-dependent aggregation of the 6HBs could be explained by *e.g.* the ability of human NfL to form homopolymers *in vitro*, [1] which in a sandwich immunoassay can cause crosslinking of the detection molecules. Solving such analyte-specific issues would be the next steps in the workflow for the optimization of the DNA origami assays for NfL detection.

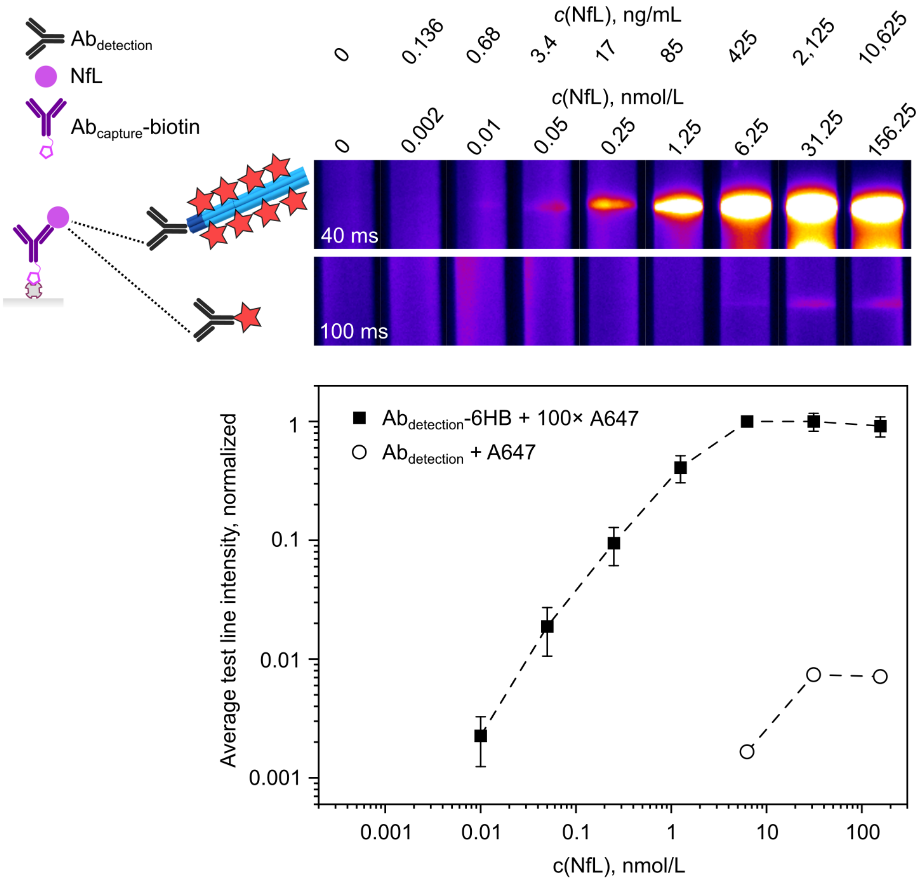

Fig. S9 NfL detection in human serum. The test strip images show the A647 fluorescence at the test line at 15 min assay time. In the top row, anti-NfL antibody-6HB conjugates with 100 A647-DNA labels per 6HB were used for the detection. For the images in the bottom row, the DNA-conjugated detection antibody was fluorescently labeled with complementary A647-labeled oligonucleotides. The concentrations of the capture and detection antibodies and A647-labeled oligonucleotides were identical in both experiments. The test line intensities are further compared in the graph in the bottom panel. In case of 6HB detection, the experiment was repeated in total three times and the intensities are presented as the average ± standard deviation. For the unamplified test strips, the result from a single experiment is shown. All test line intensity values were obtained as integrated pixel intensities from fluorescence images captured using a 40 ms exposure time. The exposure times used for capturing the strip images in the top part of the figure are shown in the bottom-left corner of each data set; due to the weak fluorescence intensity of the test line and low signal to noise ratio in the unamplified data set, 100 ms exposure time was used for improving the image quality.

Note 9: Screening of antibody pairings for cTnI detection with the A647 fluorescence assay

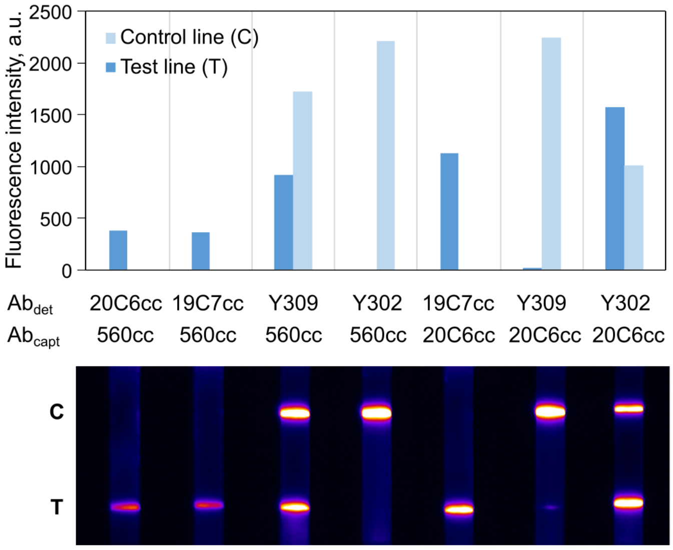

Fig. S10 cTnI detection with different antibody combinations in the A647-6HB fluorescence assay. The top panel shows test and control line intensities obtained with the antibody combinations listed in the middle panel for the detection of 1 nmol/L cTnIC in serum after 10 min assay time. On all test strips, the test line (T) contains streptavidin and the control line (C) anti-rabbit antibodies, and the direction of the lateral flow is from bottom to top. Ab_{det} refers to the DNA-labeled detection antibody in the antibody-6HB conjugate, and Ab_{capt} to the biotinylated capture antibody. All antibodies are monoclonal antibodies purchased from HyTest Ltd. and their nomenclature is according to the identifiers provided by the manufacturer. The corresponding fluorescence images of the test strips are presented in the bottom panel. Ab-6HB conjugates were formed by incubating 6HBs with a 6-fold molar excess of different DNA-labeled Ab_{det} and with 100-fold molar excess of A647-DNA labels. The LFIAs were run using the protocol described in the main text. Out of the presented Ab_{det}, Y309 and Y302 are rabbit IgG antibodies and thus captured by the anti-rabbit antibody at the control line, all other antibodies are mouse IgG antibodies.

Note 10: Comparison of the sensitivity of DNA origami signal amplification to commercial cTnI lateral flow assays

In Figure 4 of the main text, the sensitivity of cTnI detection with DNA origami signal amplification is compared to antibody-gold conjugates (Ab_2-gold), which were produced in house using a passive adsorption method commonly used in commercial LFIAs. For a further comparison to state-of-the-art lateral flow tests, commercial cTnI lateral flow tests from two different manufacturers were tested with spiked human serum samples (see Supplementary Methods) and their performance was compared to the DNA origami assays with gold-DNA labels.

The assay result for commercial tests A and B are shown in Fig. S11a and S11b, respectively. Both tests were run with two different sample volumes: the volume recommended by the manufacturers (50 μL for test A and 75 μL for test B) and the volume used by us in the DNA origami assays (5 μL). As seen in the photographs of the test strips, lower concentrations of cTnI can be detected by applying a larger volume of sample per strip, as this increases the number of analyte molecules per strip. When the test line intensities are compared in terms of the total number of cTnI molecules per LFIA (Fig. S11a and S11b, right panel), thus taking into account that both the concentration and the volume of the sample affect the test line intensity, the sensitivity of both tests is effectively identical regardless of the applied sample volume. The dependence of LFIA sensitivity on the sample volume has also been acknowledged in other studies [8].

In Fig. S11c, the commercial tests are further compared to cTnI detection with two different detection antibody-DNA origami conjugates (Ab_1-6HB and Ab_2-6HB) and in-house produced antibody-gold conjugates (Ab_2-gold). The same data for Ab_1-6HB, Ab_2-6HB, and Ab_2-gold are also shown in Figure 4b–c in the main text. The comparison shows that the sensitivity of the Ab_2-gold conjugates is equal or better than the commercial tests, justifying their use as a state-of-the-art reference on our test strips. Furthermore, the Ab-6HB conjugates outperform all state-of-the-art conjugates by ∼50–100-fold higher sensitivity.

Consequently, these results also suggest that the LoD of our DNA origami -amplified LFIAs (currently at 1.5 pmol/L for 5 μL of sample) could be further improved by using a test strip design that enables using larger sample volumes.

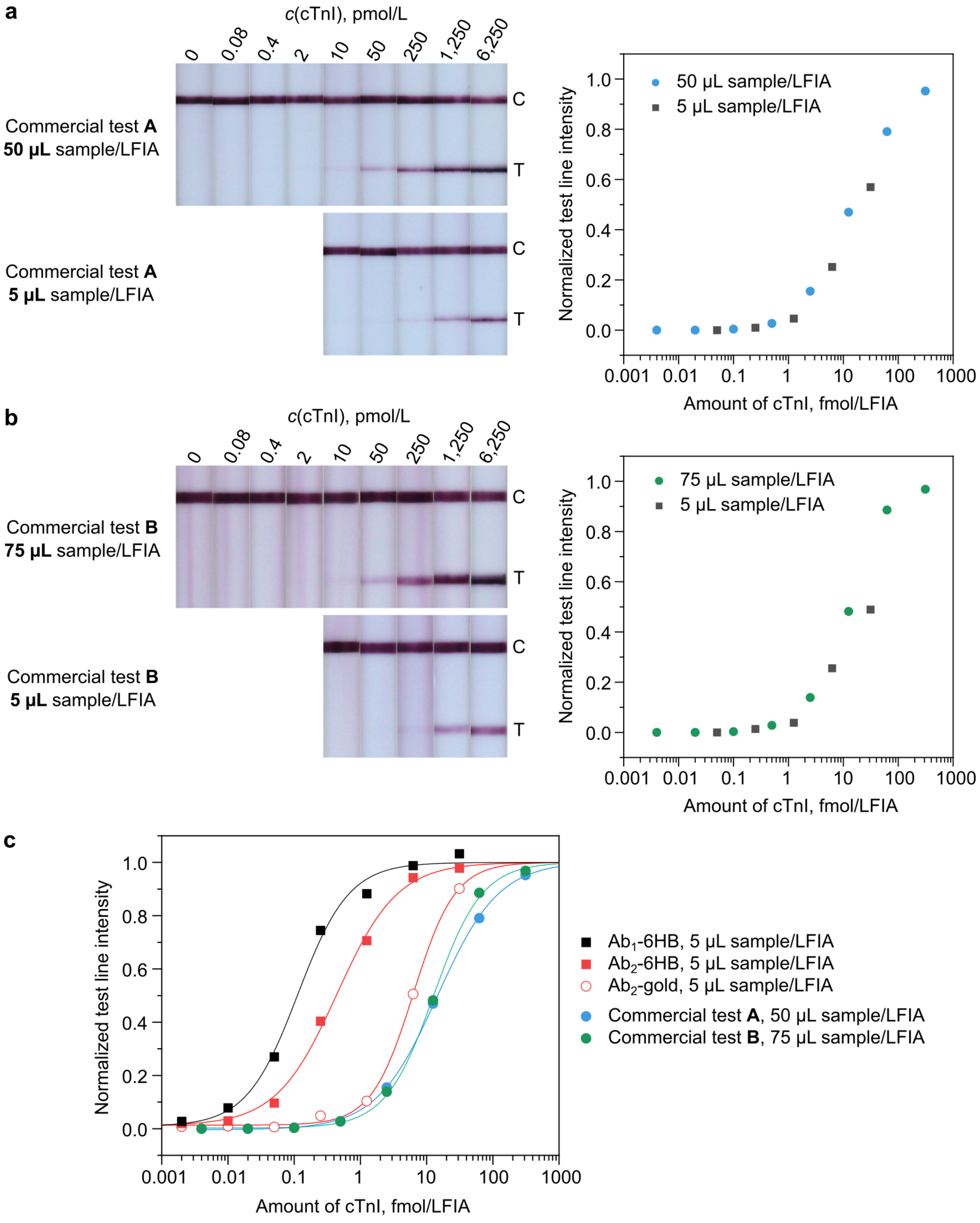

Fig. S11 Comparison of the sensitivity of state-of-the-art cTnI LFIAs and DNA origami signal amplification. **a**. Assay results for commercial test A. The left panel shows photographs of the test strip membranes at 10 min when using either 50 μL (top panel) or 5 μL (bottom panel) of serum sample per test (spiked with the indicated concentration of cTnI). In the right panel, normalized test line intensities from the photographs are plotted against the number of cTnI molecules per test, thus taking into account both the concentration of cTnI and the volume of the sample. **b**. Assay results for commercial test B. **c**. Comparison of test line intensities obtained with Ab$_1$-6HB, Ab$_2$-6HB, and Ab$_2$-gold conjugates (5 μL sample), and with the two commercial tests (50 and 75 μL sample).

Note 11: Supplementary data for cTnI LoD determination

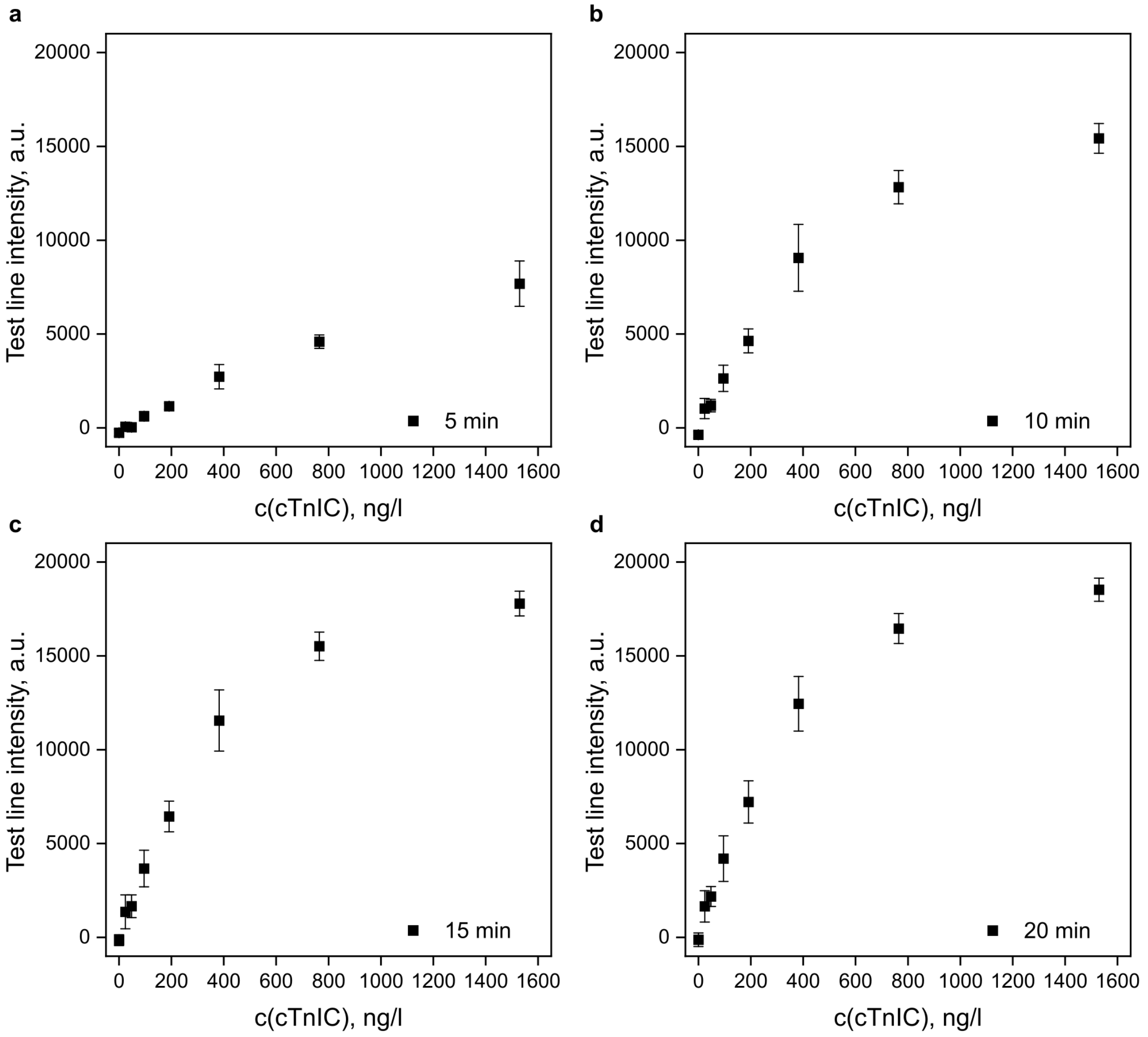

Fig. S12 Average test line intensities after different assay times in the experiment for determining the LoD of cTnI detection with Ab_1-6HB and gold-DNA labels. All data are shown as the mean ± standard deviation ($n = 3$ independent experiments). **a**. 5 min assay time. **b**. 10 min assay time. **c**. 15 min assay time. **d**. 20 min assay time.

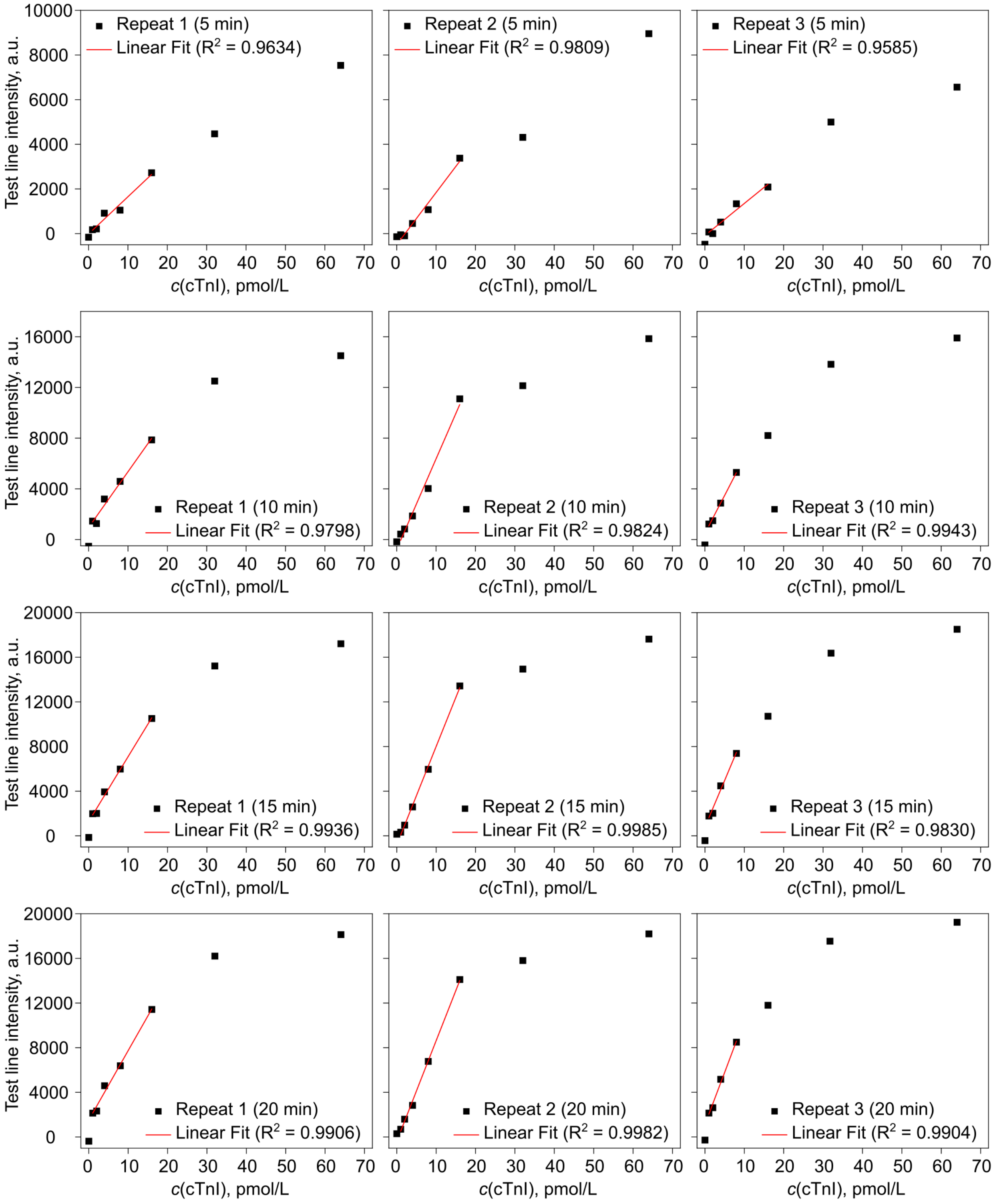

Fig. S13 Test line intensities and linear fits to the data used for determining the LoD of cTnI detection with Ab_1-6HB and gold-DNA labels. Each time point (from top down: 5 min, 10 min, 15 min, 20 min) and each of the three repeats is presented separately, accompanied by the linear regressions fitted to the data. For repeats 1–2, intensity values between 24–382 ng/L (1–16 pmol/L) were termed the linear response region and used in the LoD analysis. For repeat 3, the linear range was between 24–191 ng/L (1–8 pmol/L).

Note 12: The 6HB design

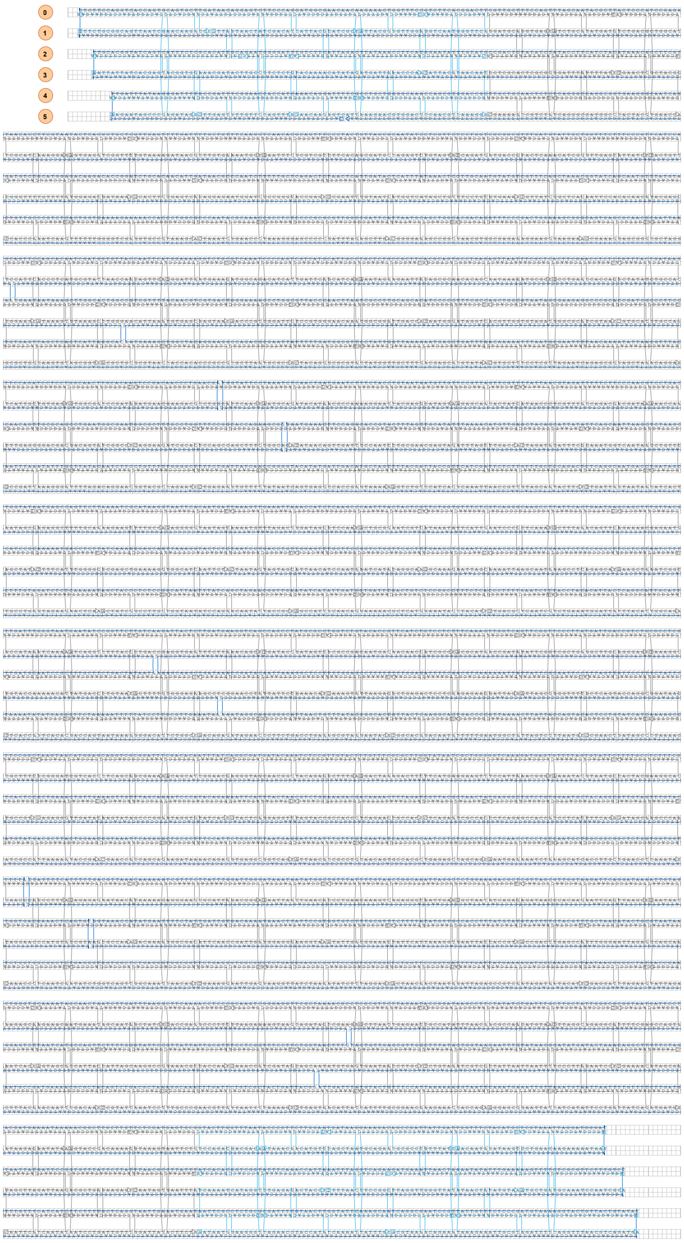

Fig. S14 The caDNAno design map of the 6HB signal amplification structure. The 6 helices are arranged into a hexagonal lattice. The p8634 scaffold is presented in blue, the staple oligonucleotides forming the binding domains in light blue, and the staple oligonucleotides forming the amplification domain in gray color.

Table S1: Staple oligonucleotide sequences for the 6HB signal amplification structure. Type 'A' staples form the amplification domain, and type 'B' staples form the binding domains.

Start	End	Sequence	Type
2[48]	1[30]	AATCCCCCTCTCTGGATGGTGGTTCCGAGTTGTTCGACTCCTG TTATCACCC	B
4[818]	2[805]	GTGCCTTGACAATAAGAGGACAGATGAAAACAACCCTCCAAA	A
1[693]	3[706]	GCTTTAAGTGAATATCTGAAATGGATTAGTAATGTAGAGCAT	A
5[133]	1[146]	TCACCCAGCAACAGCGGGTTTTGTTTTATCCATGCAAAGGAG	A
4[62]	2[49]	CGAAAATAGTCATTTGTTGTTCGCCATCCATTACGGAAGACA	B
1[1281]	3[1294]	TTTAGGCTAGCAAGCATCATATTCCTGAAATTAATGAATCCT	A
5[931]	1[944]	ATTAACAATAAATCTAGTACCGCCACCCCGTCTTTGCTTTGA	A
2[258]	0[245]	GTTTAGCCGCTCACCCCGCTTTCCAGTCAGGAAGGCATCAAA	A
3[119]	5[132]	TGAACTCTGTTTATTCTGACGCTGGCATTCTCGGATGAACCA	A
5[973]	1[986]	AAATGAAGTTGAGGCCTCAGAGCCACCAACAGACAAACGGGT	A
1[315]	3[328]	GGATTATTTTCCACACCACACCCGCCGCCTTCCTGAGTAAC	A
0[958]	4[945]	CAAGCGCAGACTTTATCTAAAGTTTTGTTCAGAACATATTCA	A
3[665]	5[678]	GGCAAAGAGCTTCAATAAATATTCATTGGAGAAACCTACATT	A
0[454]	4[441]	TTGTGCATCCGATAATTCGCCATTCAGGGACAGTAGTATAAG	A
1[483]	4[483]	AATGCAGAGATTTATATAATCAGTGAGGCATATGTACCCCGG	A
3[161]	5[174]	GCACTTCGTGCAGATGTAAAAGATAACGAATCAAATAAAGCA	A
1[1407]	3[1420]	AACAATAGAAACCATAAAGAAATTGCGTCGGGAGATATGTAA	B
5[9]	0[2]	AAGAATAGCCCGCACTGGTGACCTGGAAGAG	B
5[259]	1[272]	GCGAAAGCTCACTGAATTCCACACAACAACATTGCATTATCT	A
2[426]	0[413]	GGAAGGGCTGCCAGGTAAACGTTAATATGAGCTAAGTAAACA	A
3[413]	5[426]	CGTGCATCGATCGGGCTATATCTGCCACCTTATCAACAGGAG	A
4[1154]	2[1141]	ATAGCCCTTAGAAGTTTATCCTGAATCTAATTGAGGTTAGCA	A
4[1028]	2[1015]	GAGCCGCTCAATCATGAAAATAGTAAAACAACCCAGTCACCA	A
5[175]	1[188]	CTAAATCTATTGGGCAGGAGAATGGATCAGATTGGAAATGCT	A
3[749]	5[762]	ACTTTTGTTTATTACTGACCGAGGTGAAGAGTAATTCTGGCC	A
2[930]	0[917]	TTAGTAAGGAGGTTCTCATTAAAGCCAGGTGAGGCATCGCCT	A
3[455]	5[468]	CAGGAAGAAGCGCCGATGAACGAAGACGCAGGGTTCAGAATC	A
2[552]	0[539]	TATAATGCATTTCGAGTCTGGAGCAAACGTAGCAATTAATAA	A
3[1169]	5[1182]	AGTAGCAATGATTAAGAAACAATGAAATTAGTTGCCGACAAC	A
2[1392]	0[1379]	TTCTGACCTCCGGCTTGCTTTGAATACCTCAAAATCTTATCA	B
4[692]	2[679]	AAGATTCTCAATCGAGGCTTGCCCTGACAATCCCCCGCGTTT	A
4[356]	2[343]	AACGCCAGCGCGTAGGCAATGCATGACGTGATTCAGGGTAAC	A
5[1057]	1[1070]	TGAAAGGCCTCAGAATTCAACCGATTGATTTTGTCGACGGGA	A
1[231]	3[244]	TCAGATGACCAGGAGAACGTGGCGAGAAGGGAAACGTGAAAT	A
3[371]	5[384]	TAATGGGGGGGGATCAGCGCAACACAGCCTCAGGCCGTATAA	A
1[609]	3[622]	GAGGGGGTGAATTATGGTAATATCCAGATGATAAAAATTCTA	A
1[1239]	3[1252]	GCTTAATGAACGCGCATTTTGCGGAACAATGGAAACCTTGCT	A
3[1295]	5[1308]	TGAAAACAAGCCTGTTTTCGAGCCAGTAAGGAATCATGATGG	A
5[469]	1[482]	CTGAGAATCAGAAATCCAGCCAGCTTTCTCTGGTGTTCAACT	A
5[91]	1[104]	CGAAAAAGCAGCAACCTCGCACTCGCGGAGATATAGGTGAGT	A
1[987]	3[1000]	AAAATACCTAAAACAAGCATCACCTTGCCGCCGCCTTTCAGG	A
2[342]	0[329]	GCCAGGGCGGATTCTAATTCGCGTCTGGCGCTTAAATTTGAC	A
1[1071]	3[1084]	GAATTAATATTTATGAAGGTTATCTAAACGCCTCCAAGGTAA	A
1[651]	3[664]	ACTGCGGTAAATTGGAAAAACGCTCATGGACAGTCCAGGCAA	A
1[399]	3[412]	GCCTGAATCGAAGTTAGAATCAGAGCGGTTTGTTATCGTAAC	A
1[63]	3[76]	ATTACGGAGTTTCTAAAGAACGTGGACTTTTGCCCAACCCAA	B
4[608]	2[595]	CGGAGAGGCCTTGCCCTTATGCGATTTTAAGAAGTTTTTGAT	A
2[594]	0[581]	AAGAGGTTTGGGGCATTTTTGAGAGATCGTAGAAGGGCTCAT	A
3[490]	5[510]	TATAACAGTTGATTTCTGGAAACGCCAAAAGGAATGGTAGAAG TAAAAG	A
4[1446]	0[1421]	ATATACAGTAACAGTACCTTTTACATAGATTTTTTTACGA	B
5[889]	1[902]	CGAACCAATTTACCAAGTATAGCCCGGATTGCTAAGCGAAAG	A
1[1365]	3[1378]	ATAAACAAACGGGTTAGAACCTACCATAAAGTTACACTACCT	B
5[1225]	1[1238]	TTGAGTAATCCGTACAGCAATATGTGAGTACAAAGCCGAAGG	A
1[2]	5[27]	TTTCTGCGGCAGTTAATCGAACAAGAAGCACTGAGATAGG	B
3[1253]	5[1266]	TCTGTAATATAAAGCGCCATATTTAACAAAGGCTTCACCAGA	A
0[1042]	4[1029]	TTTAACGAATAACATGGTTTACCACACTCCGTAAGACCCTCA	A
2[636]	0[623]	ACAGGTCTAGTAGCTCAACCGTTCTAGCACAATATAACTTTA	A
1[903]	3[916]	ACAGCATTGTGTCGAAGATAAAACAGAGAATGGAATATCACC	A
5[301]	1[314]	ACGCTGCTCGTGCCCTGGGAACATTAAAAAAACGACAGTTCA	A

1[1155]	3[1168]	CAATAATCTACAATTATTAGACTTTACACGGCATTAATCACC	A
0[1252]	4[1239]	ATTCTAATGAGAATCCAACGCTCAACAGTGAATAACAGTACA	A
5[763]	1[776]	AACAGAGTTTTATTAGCCTCGGAACCTAGTATCGGAACAGCT	A
4[776]	2[763]	TGCCTATATAGAACCTGACCTTCATCAATTTCTTATTTATCA	A
5[637]	1[650]	AGCCATTATGATATATTAACATCCAATAGGAAGCAGTCCAAT	A
2[1056]	0[1043]	ATAGAAAGGGCGACACCGCCACCCTCAGGGCAAATTTTTTTG	A
2[1098]	0[1085]	AACGCAACGGAAATGCCACCACCGGAACATATCTTATAAACA	A
0[790]	4[777]	GGCGCATGATAGTTAGGAGCCTTTAATTTTATTCTTGCCCCC	A
2[174]	0[161]	CAATGTTCGAGTCACGCCAGGGTGGTTTGGTGCCGACTCAAC	A
2[300]	0[287]	TGCCAAGGTAAAGCTAATGAGTGAGCTACAAGTGTTGTGCTG	A
0[1294]	4[1281]	CGCCCAAAGAGGCATTTAGTATCATATGTCCCTTATACATTT	A
4[440]	2[427]	CAAATATAAAGGGAAGGAGGATCTGGAATCATTGTACTGTTG	A
4[146]	2[133]	GAGACGGAATCAAGTTACGAAGGTGTTATCGCATCAGACATC	A
5[1309]	1[1322]	CAATTCAAAACAAATAGCTTAGATTAAGCGGAATCAATATAA	A
0[202]	4[189]	AAACTGCGCGTCGTGAGTCTGAGCAAAACCCGGGTAGAGGCG	A
4[1070]	2[1057]	CCGCCACAATTGAGCCCAATCCAAATAACGCATTAACAATCA	A
3[1379]	5[1392]	TTTTAACCTAAATTCAGCTAATGCAGAAGTCTTTCTATTTGC	B
4[650]	2[637]	CATCAATGCAACAGGGCTTGAGATGGTTGGATAGCAACTCCA	A
4[902]	2[889]	TCTCTGACCAGCAGAAATCCGCGACCTGCTCAGCAACAACTT	A
0[1000]	4[987]	GAATACAGTAATGCGCCTGTAGCATTCCCCCTCATAGCATTG	A
1[777]	3[790]	TGATACCAGGCTGGCCTTCTGACCTGAACAGTTAAGAAACAT	A
5[1393]	1[1406]	ACGTAAACGCCTGATTAGGTTGGGTTATTAGTTAAGTTTATC	B
4[1364]	2[1351]	CGCAGAGGAAGGGTATTAAACCAAGTACTCCAGACGTGTGAT	B
3[791]	5[804]	GAAAGTACTCCAAAGCGCCGACAATGACCGGTGTAGAATACG	A
3[833]	5[846]	ATTAGCGCGAATAATATTCGGTCGCTGATAAGGGATTAATGC	A
5[847]	1[860]	GCGAACTCAGGAGTGCTCAGTACCAGGCAAGGAACAGGGAGT	A
5[1099]	1[1112]	ACTAACAAACCAGATATTCATTAAAGGTCAACATAAATTGAG	A
4[1196]	2[1183]	TTAGCGTAAATCCTCCTTAAATCAAGATAGCAATACCAAAAG	A
0[706]	4[693]	TCATTCAACAGTTCAGCCCGAAAGACTTAAGCCTCGTAGGTA	A
3[1211]	5[1224]	GAAACCACCGAGGATTAAGAAAAGTAAGACCTCCCTAAAAGT	A
0[370]	4[357]	GAAGCGGAGAATTAGTGCTGCAAGGCGACGGCGGACAATAGG	A
2[216]	0[203]	GTATCAACGTAATCTGAATCGGCCAACGGATTTAGAGTAGGG	A
5[343]	1[356]	CTACAGGTCAAAAATCCGTGGGAACAAATTAAGTTGCATCAG	A
3[917]	5[930]	GTACTCAATGAATTAGGGTAGCAACGGCTTGTATCGGTCAGT	A
4[482]	2[469]	TTGATAAGTGTTTTGGAATACCATCGTTCCTGGCACCGGAAA	A
2[720]	0[707]	AAAGATTAATCGGTAATGCAATGCCTGATTTACATAAGCTGC	A
1[1323]	3[1336]	AGTACCGAGCCGTTAATCCTGATTGTTTCCTGAGCGAAGAGT	A
3[77]	5[90]	TATTACAATATTCCGGTCAATGGGTTCAAAGTCCGCAAAGGG	B
1[735]	3[748]	ATCAGGTGATATTCGACCAGTAATAAAAAAAATTTCTGTAAT	A
0[538]	4[525]	AACGAACCACTATCTCAACATGTTTTAAATTTAGTTCGATGA	A
0[664]	4[651]	CGAGTAGAATCGTCAAGCGAACCAGACCAATCATAAAATCAC	A
0[622]	4[609]	ATCATTGTAATAGTGAGAGTACCTTTAATGGCATCTTAATGC	A
3[245]	5[258]	TGTTATCTGAAACGTACTCAAACATCGGAGCAATAGAAGAAA	A
0[286]	4[273]	GAACTGCTCCATTCATTCGTTTACATAATACGAGCTTAATTG	A
4[860]	2[847]	GATGATAGATAGCCCAGACGGTCAATCAGGCTTGCAACTAAA	A
0[118]	4[105]	AAGCGTATATGATGGTAGATGAAGGTATGGATTTAGCCCTGA	A
0[328]	4[315]	GCAGACCATGAAAGAGTCACGACGTTGTTGTGAGCGTAGCCA	A
3[539]	5[552]	TTAGATACTGTAGCATAACCCTCGTTTAATCTACGTACTTCT	A
3[287]	5[300]	ATAAAGTCTTTATTACCCTCATGCGTATCATCGATAGCGGTC	A
5[1267]	1[1280]	AGGAGCGTCATTTGCTATTAATTAATTTCGTTATACATGTAA	A
4[1322]	2[1309]	GATGATGTCAATATTTTATTTTCATCGTATAAGAGATAATTA	A
4[398]	2[385]	CATTAAATCCTCGTGAGCGAAATTAACTAATAAAAGCCAGCT	A
3[959]	5[972]	CTCAGAACGTAACGTTCATGAGGAAGTTCCCAGCGCAGCAGC	A
2[510]	0[497]	TACGGTGCCCAATTACTAGCATGTCAATCCACCGAAGATTCA	A
2[1182]	0[1169]	AACTGGCCCATTACTAGCGCGTTTTCATAACAATTTATTTTG	A
0[76]	4[63]	GGAAACCGGTTGGAATGAAGGTTTATAAATGATTACAGCAGG	B
4[314]	2[301]	GCTTTCAGCGTAACTGAATTGGTAACACTAACCAACGGCCAG	A
0[874]	4[861]	GCCGGAACGCTTTTGGAGTGAGAATAGAGGATAAGGGCTTTT	A
3[1043]	5[1056]	GACAAAAATTCATATAAAAACAGGGAAGGAAACGACAACAGT	A
4[230]	2[217]	GCCAGCTAGCCGGCGAACGAGGATATTGCAGAGTCTAAATGA	A
3[1421]	1[1439]	ATGCTGAAAAACTTCCTGAACAAGAAAAATAAT	B
1[1029]	3[1042]	CAGAGAGTCAAAAAATATCTGGTCAGTTAGCCACCCGCCAAA	A
0[1336]	4[1323]	ACAAGCAACAAAAGATAAGAATAAACACACGCTGAAAAAGAA	A
0[412]	4[399]	GAGAGGTCCACCAGTGCGGGCCTCTTCGGGGCGCAAAATTCG	A
2[972]	0[959]	TAGTTAGCCGCCACCAGGTCAGACGATTTGAGAGCATTATAC	A
0[580]	4[567]	TATACCAAATAGCGGCGGATGGCTTAGACCTGTTTGCTATCA	A

2[1308]	0[1295]	CTAGAAAATAGCGACATCAAGAAAACAATTATCAGATTACCG	A
1[189]	3[202]	GAATTTCGTGTTGATAAAGGGAGCCCCCCGCGGGGACCGAGC	A
0[496]	3[489]	TCAGTTGATACATAGTTTCATTCCCGCTCGGCACA	A
2[1266]	0[1253]	TTACCAGATCGTCGAATTACCTTTTTAAAGAAACATCCGGT	A
0[1378]	4[1365]	TTCCAAGACATGTTTAATGGTTTGAAATCTGAGAGAAAATCG	B
3[875]	5[888]	GAGAGGGTTTCAGCGCGGGATCGTCACCCTCCATGTACCGAA	A
2[1443]	3[1443]	AGACAAAGAACGCGAGTGCAAATCCAATCGCA	B
4[1280]	2[1267]	AACAATTGAATTATCAAATCAGATATAGACGCCAACAAATTC	A
4[104]	2[91]	GAGAGTTCCGTCTAACTGGAAAGCAACGGGATGCAGAGTCGG	A
2[888]	0[875]	TCAACAGTTGATATGTTCCAGTAAGCGTTTAAAAATTACTTA	A
0[1168]	4[1155]	CACCCAGAAGAGCAAGACTCCTTATTACCCAGCAATTCGGTC	A
0[1420]	4[1407]	GCATGTAGATAAGTTTTCAAATATATTTATAACTAAACAATA	B
5[28]	2[38]	GTTGAGTAATCGGCAAACGATACTTGCCATTC	B
1[105]	3[118]	ATCTGCATTGCTAATCAGGGCGATGGCCCCGCCTGTTTTATC	A
0[1126]	4[1113]	AGCGTCTATCAGAGATACATAAAGGTGGGAATTATATCAAAA	A
0[1084]	4[1071]	GCCATATCTGAACAACGGAATAAGTTTAGGGAGGGCTCAGAG	A
5[385]	1[398]	CGTGCTTTTTTTGTACGTTGGTGTAGATCTATTACATGCGCC	A
2[37]	5[48]	TGCGTAACTATCGACATCTGGGAACAGTTTG	B
2[1224]	0[1211]	AAGGAAATCGATAGATCAGTAGCGACAGTTAATTTGACTTGC	A
1[567]	3[580]	AAACCAAGTCAGGACATCACTTGCCTGATACAAAGAGCTATA	A
1[861]	3[874]	TAAAGGCCGAGGCGCTAAAACATCGCCACATACATTGCCGTC	A
1[525]	3[538]	AGAGCAATAACGGAGCAAATTAACCGTTAAGAGAATTGACCA	A
5[49]	1[62]	GAACAAGCCTGTTTTACAAAACATATAGGTCTAATCATCGCT	B
1[273]	3[286]	TACTGTTTGGATGACGCTAGGGCGCTGGACTCACACGGAAGC	A
5[217]	1[230]	CGGGGAAGCATTAAATGGTCATAGCTGTGAGTTTATGTAGTG	A
3[203]	5[216]	TCGAATTTGAGTTACTTCACAGCGATGCCGCTAACAGCTTGA	A
0[1439]	5[1446]	ATCCCATCCTAACAGGTTTAACGTCAGATGA	B
4[1238]	2[1225]	TAAATCAACATTATAGGCGTTTTAGCGACAGATAGTTACCAG	A
5[1141]	1[1154]	TGAGGATCCTTATTTTTGGGAATTAGAGGCAGTATTTAAGCC	A
1[945]	3[958]	GGACTAAGAAACAACAACAGTGCCACGCGGCCTTGCGCCACC	A
2[804]	0[791]	AAAAAGGTTAAGAGAGTGCCCGTATAAAAGCGTAACAGACCA	A
4[1406]	2[1393]	ACGGATTACAGAAAATCAATAATCGGCTCGCGCCTTTTCATC	B
5[553]	1[566]	TTGATTAGCCTGAGCAAATGGTCAATAAGCTTAATACGATAA	A
2[1350]	0[1337]	AAATAAGGAATTTAATTCATTTCAATTAGGATTATATCGAGA	A
0[160]	4[147]	GAGCAGCTCGGTTTTCCGGTGTCTTGTCTGGAGATCACCAGT	A
2[468]	0[455]	CCAGGCAATCGCACAGCCCCAAAAACAGGGTACGCGTCGGAC	A
2[90]	0[77]	CATACAATAACAATGCGGTCCACGCTGGCCAACGTTGAAGAC	B
5[1351]	1[1364]	AATAATGGCGAATTTCAAAATCATAGGTACCGACCGACGACA	B
5[595]	1[608]	ACTATCGGGTAGCTGCGAGCTGAAAAGGTTGCTCCTTTGCCA	A
3[1337]	5[1350]	CAATAGTGCGTTAAGTAAAGTAATTCTGCGCACTCACTTCTG	A
4[944]	2[931]	CAAACAACCGCCTGAGTACAACGGAGATTACAGAGCCAGACG	A
3[329]	5[342]	AACCCGTTTTTCCCATGGCAGACATCATACTGGGGTGCGCCG	A
5[1015]	1[1028]	CAAACCCCACCAGATAGGAACCCATGTAGAGTTTCGCCTTTA	A
4[566]	2[553]	GGTCATTGTAATAACGTTGGGAAGAAAACCAGACGTGCTGAA	A
3[707]	5[720]	AAAGCTAAAGAGGAAGAAAACGAGAATGCGTAACATGGCAGA	A
1[1113]	3[1126]	CGCTAATTTCCAGAGATTAGAGCCGTCATTTCATACACCGTC	A
1[441]	3[454]	GTGGCGAAGTTGCCTTTTAGACAGGAACGAAGATTTCGGCCT	A
3[623]	5[636]	CTAATAGAGGATTAAAAATGTTTAGACTTAATTTCTACCGCC	A
4[986]	2[973]	ACAGGAGAAATCTAACTCATCTTTGACCTCCATTAGCCCTCA	A
2[132]	0[119]	ACGAAGGGCTACGGCTGATTGCCCTTCACACTACGTGGGAGT	A
3[5]	2[5]	AATTATTATCACGAGTGACTGATACCGTGCAA	B
2[762]	0[749]	GCTTGCTCGGGAGATCAACGCAAGGATAGGGACATCTTGACA	A
4[524]	2[511]	ACGGTAACCATCACACAACATTATTACATACGAGGACTAAAG	A
3[1001]	5[1014]	GATAGCACTACAACCACTACGAAGGCACCGAAAGACAAATAT	A
1[1197]	3[1210]	TACCGAATTTGAAGTTGCCCGAACGTTAAATCAAGCACCAAT	A
5[721]	1[734]	TTCACCATATTTTATGTACCAAAAACATGCGGATTATCAAAA	A
2[384]	0[371]	GGCGAAAATAGGTCTAAATCAGCTCATTACGAGCAACTGCGT	A
0[748]	4[735]	AGAACCGCTTTACCTAGTCAGAAGCAAATATGACCTTAGAAC	A
2[846]	0[833]	GGAATTGGGGTTTTGTACTGGTAATAAGTTAGTCTACCGAAC	A
4[272]	2[259]	CGTTGCGGAGCGGGACGGGAAAGAAACCGTTGAGTTGATACC	A
3[1085]	5[1098]	ATATTGAAGACACCCCCTGAACAAAGTCTTACAAATAGGAGC	A
5[1183]	1[1196]	TCGTATTCAGACTGCATTAGCAAGGCCGGGAATACGCTATCT	A
3[1127]	5[1140]	ACCGACTAAAATACAGATAACCCACAAGTACCAACATACATT	A
5[511]	1[524]	AGTCTGTTCGTAAACTGCGAACGAGTAGATATGCACATAGTA	A
0[832]	4[819]	TGACCAAACCGATATAATTTTTTCACGTAGAGAAGGGGGTCA	A
1[357]	3[370]	AAATAGACAGAGCACTATGGTTGCTTTGTTTTAACTTGACCG	A

1[819]	3[832]	ACGCATACTTTGAATTTTTGAATGGCTATTTTAACGATTAGG	A
1[147]	3[160]	AGTGAGAGTGAGTATTTTTTGGGGTCGATTCTTTTGATAAAT	A
4[734]	2[721]	CCTCATAGTCACACATTACCCAAATCAAACCATAAGCATCAA	A
4[188]	2[175]	GTTTGCGGGAACCCGCTTGAAACAGCAACTTGTGAGCGTTAT	A
3[581]	5[594]	TTTTCATCATTTTTAGAGGCTTTTGCAAAAGAACTAACTCAA	A
2[678]	0[665]	TAATTCGAATTAGCTGAGAAAGGCCGGAGAAATACACCAGAA	A
0[916]	4[903]	GATAAATCGGAACGTTCTGTATGGGATTATAGGTGAGCGCAG	A
2[1014]	0[1001]	GTACAAAAGCCCAAACCACCACCAGAGCTGAACCTGGCAAAA	A
0[244]	4[231]	CGCCGCGATGACCGACATACATTGCAAGTTCCTGTCTGTCGT	A
5[805]	1[818]	TGGCACAGAGTAACGCTGAGACTCCTCATGAAAATATCGCCC	A
5[427]	1[440]	GCCGATTTTAAATTTTTGAGGGGACGACCTGCGCATGTGAGT	A
0[1210]	4[1197]	GGGAGGTGCCCTTTAACGCAATAATAACGAAACGTTTTGCCT	A
4[1112]	2[1099]	TCACCGGACTAATAGCCTAATTTGCCAGAGAGGGTTAAAAGA	A
1[31]	4[9]	GTTAAATGTGCGAGAAATACGGTGGAAAATCCCTTATAAATCA A	B
5[679]	1[692]	TTGACGCAAAAGGGAAAATTAAGCAATACAAATATCTCAAAT	A
2[1140]	0[1127]	AACGTAGTGAGCCAAGCGTTTGCCATCTATAGATAGCTAACG	A

Note 13: cTnI LFIAs with different antibody-origami ratios

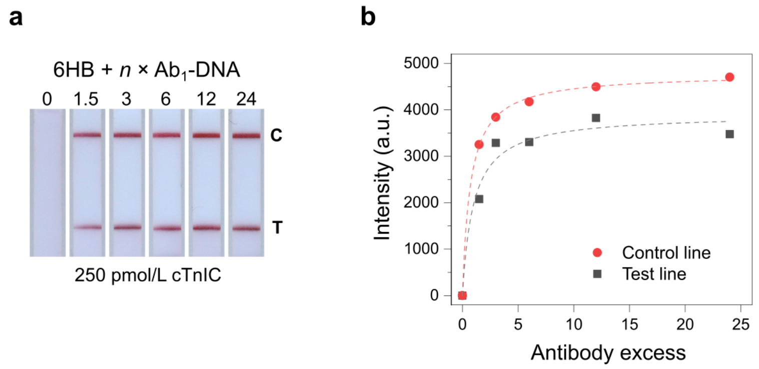

Fig. S15 The effects of changing the molar ratio of Ab_1-DNA to 6HBs in the Ab_1-6HB self-assembly reaction on LFIA performance, tested by the detection of 250 pmol/L cTnIC in serum using gold-DNA labels. **a**. Photographs of LFIA test strips after 12 min assay time. The test strips were run using the gold-DNA running protocol described in the main text. All test strips contain the same concentration of the biotinylated $Ab_{capture}$. The direction of the lateral flow in the strip images is upwards. The control line (C) is anti-rabbit antibody and the test line (T) streptavidin. **b**. The test and control line intensities of Fig. S11a plotted against the antibody excess.

Unprocessed Gel Scans

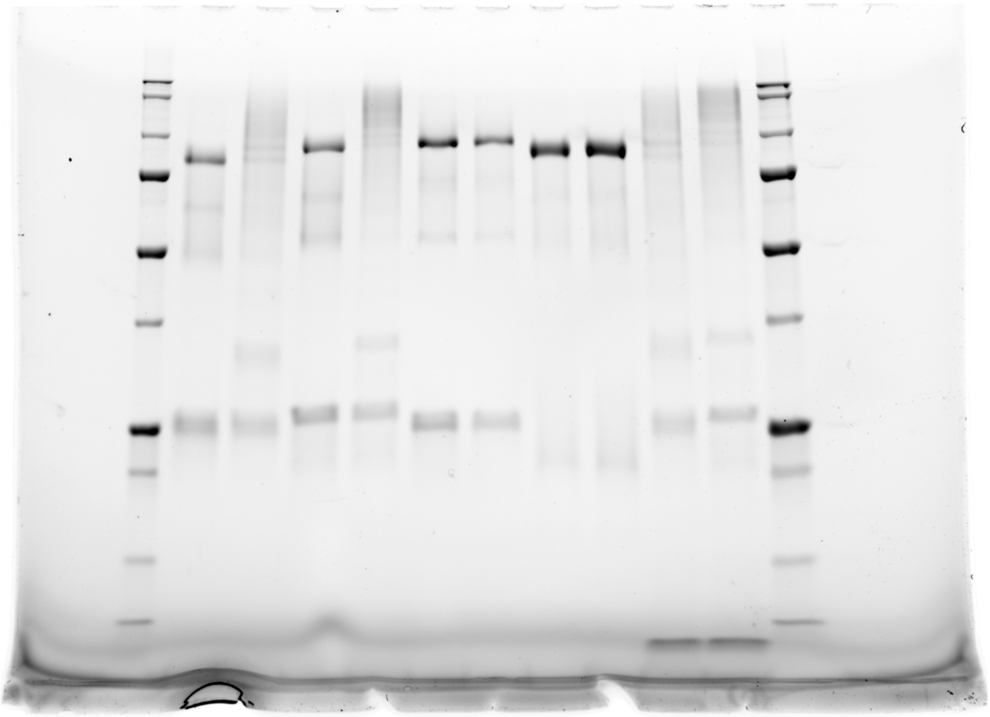

Fig. S16 Uncropped SDS-PAGE gel scan (protein stain) used in Fig. S1a.

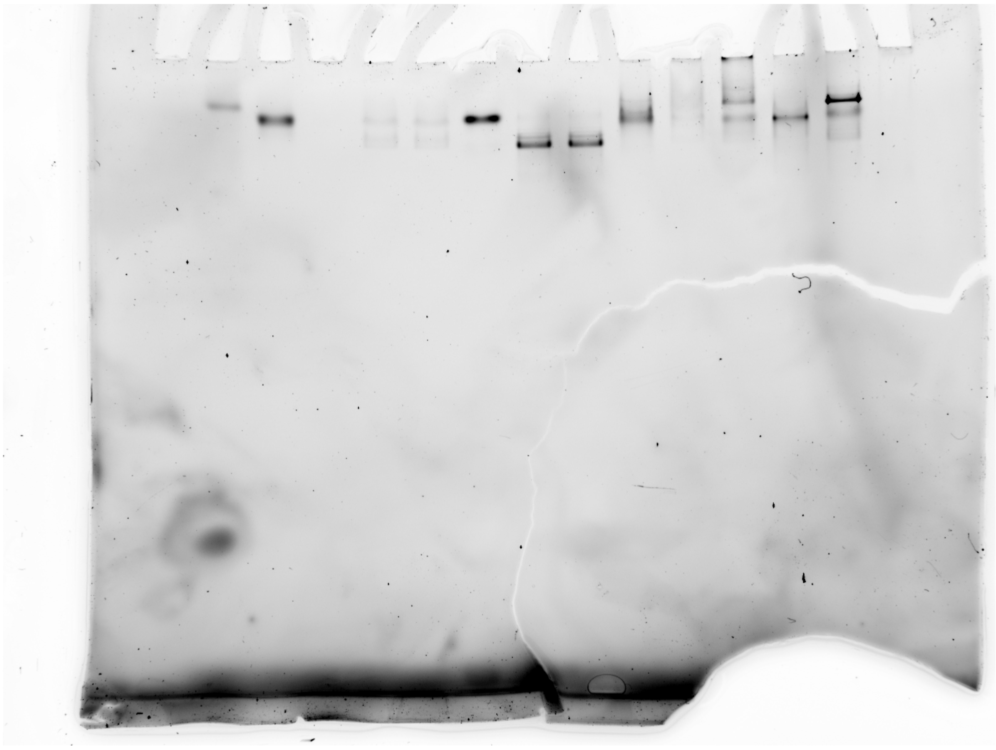

Fig. S17 Uncropped SDS-PAGE gel scan (protein stain) used in Fig. S1b.

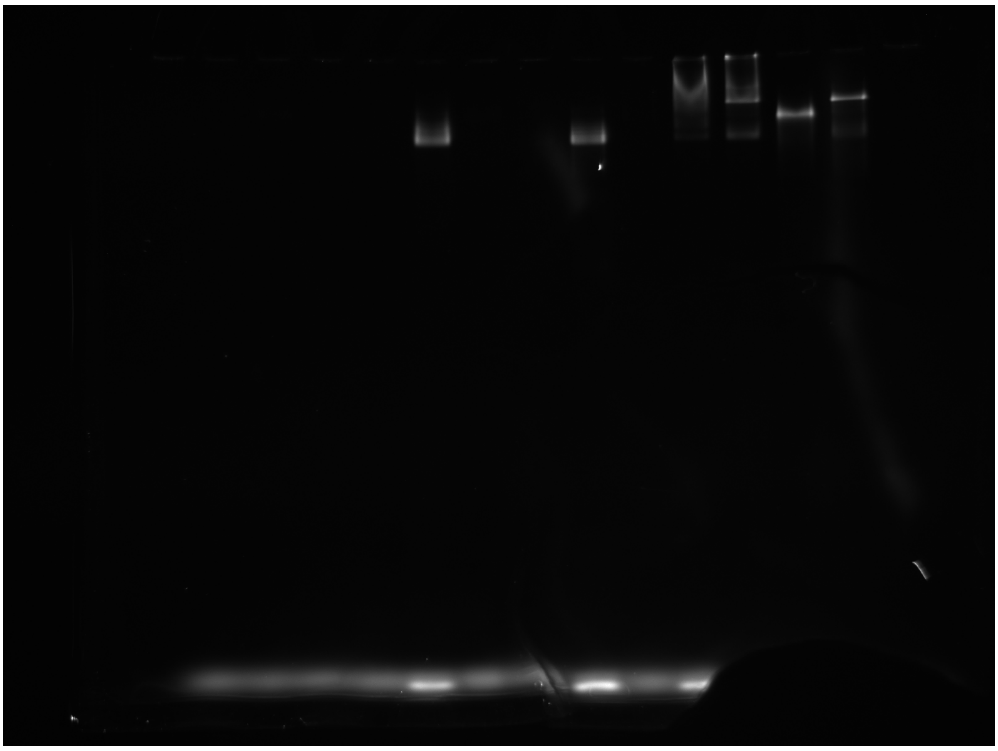

Fig. S18 Uncropped SDS-PAGE gel scan (Alexa Fluor 647) used in Fig. S1b.

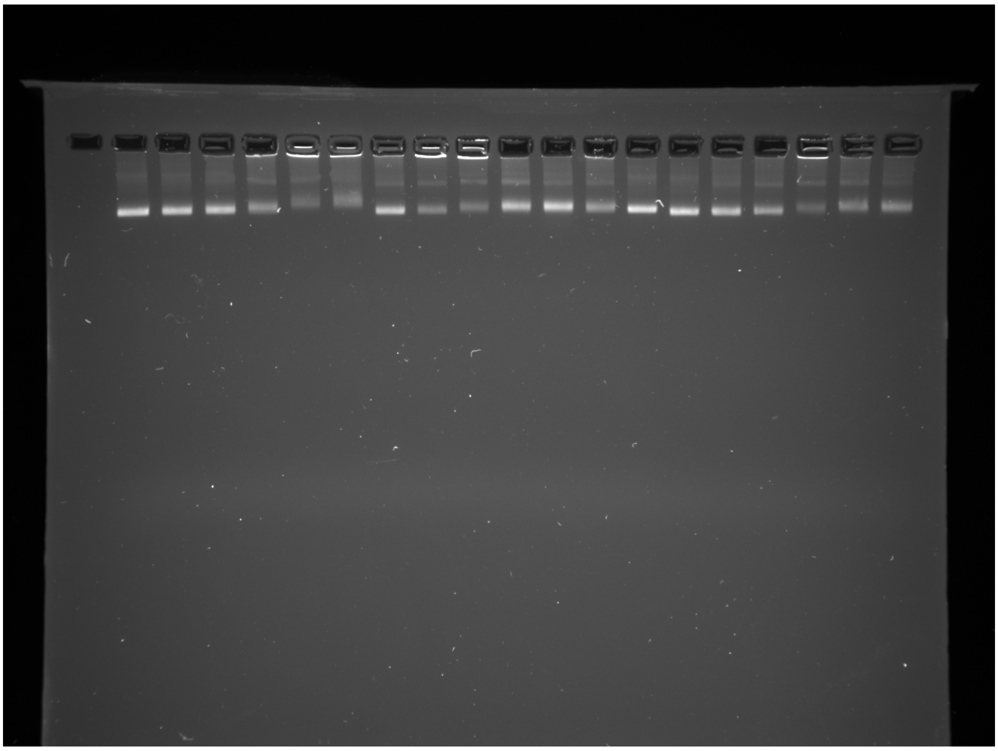

Fig. S19 Uncropped AGE gel scan (Sybr Safe staining) used in Fig. S2.

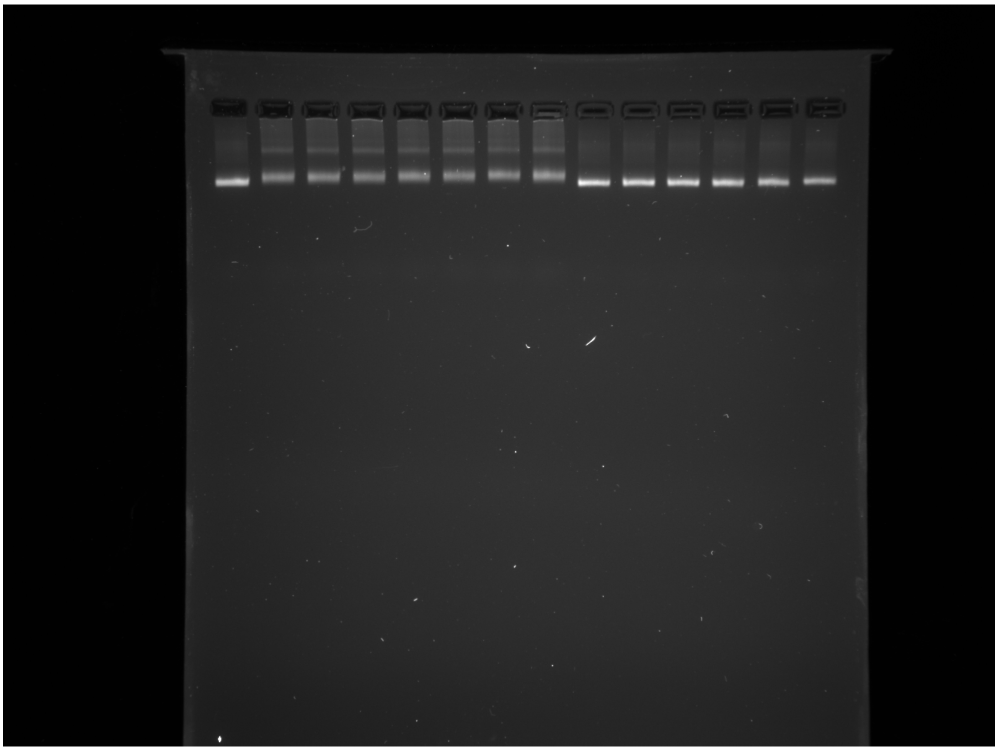

Fig. S20 Uncropped AGE gel scan (Sybr Safe staining) used in Fig. S6.

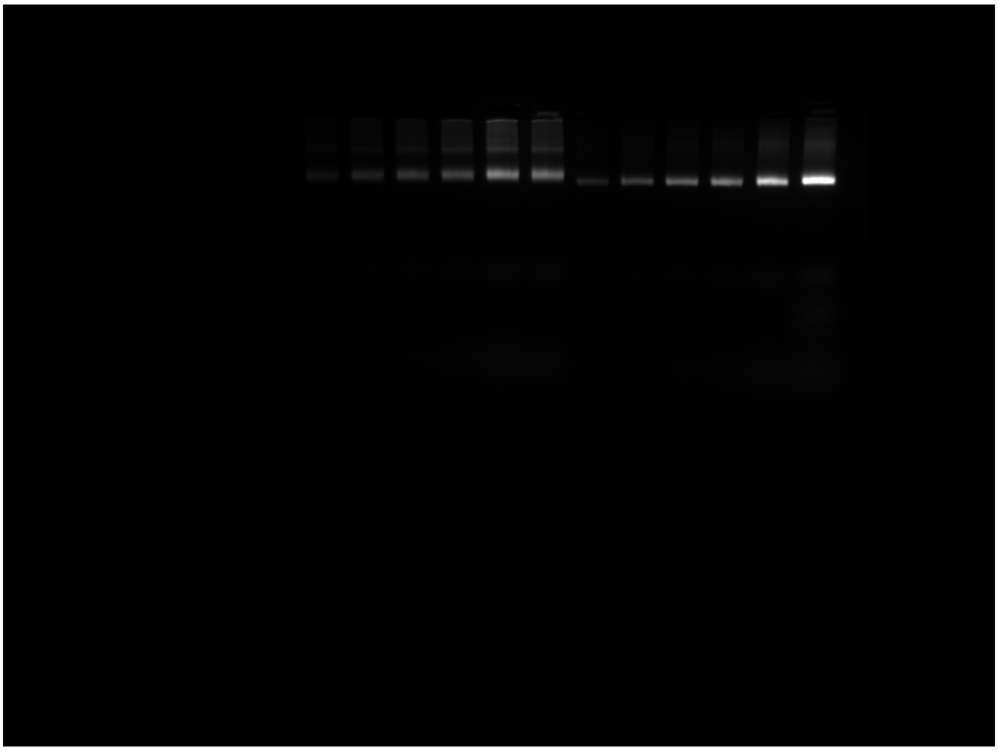

Fig. S21 Uncropped AGE gel scan (Alexa Fluor 647) used in Fig. S6.

Supplementary References

[1] Yuan, A., Rao, M.V., Nixon, R.A.: Neurofilaments at a glance. Journal of cell science **125**(14), 3257–3263 (2012)

[2] Yuan, A., Nixon, R.A.: Neurofilament proteins as biomarkers to monitor neurological diseases and the efficacy of therapies. Frontiers in neuroscience **15**, 689938 (2021)

[3] Alagaratnam, J., Widekind, S., De Francesco, D., Underwood, J., Edison, P., Winston, A., Zetterberg, H., Fidler, S.: Correlation between CSF and blood neurofilament light chain protein: a systematic review and meta-analysis. BMJ neurology open **3**(1) (2021)

[4] Truffi, M., Garofalo, M., Ricciardi, A., Cotta Ramusino, M., Perini, G., Scaranzin, S., Gastaldi, M., Albasini, S., Costa, A., Chiavetta, V., *et al.*: Neurofilament-light chain quantification by Simoa and Ella in plasma from patients with dementia: a comparative study. Scientific Reports **13**(1), 4041 (2023)

[5] Zhang, C., Deng, F., Li, Y., Hall, T., Goldys, E.: Paper-based lateral flow assay for the point-of-care detection of neurofilament light chain. In: 2023 45th Annual International Conference of the IEEE Engineering in Medicine & Biology Society (EMBC), pp. 1–4 (2023). IEEE

[6] Ahn, J.W., Hwang, J., Lee, M., Kim, J.H., Cho, H.-J., Lee, H.-W., Eun, M.-Y.: Serum neurofilament light chain levels are correlated with the infarct volume in patients with acute ischemic stroke. Medicine **101**(39), 30849 (2022)

[7] Sanchez, J.D., Martirosian, R.A., Mun, K.T., Chong, D.S., Llorente, I.L., Uphaus, T., Gröschel, K., Wölfer, T.A., Tiedt, S., Hinman, J.D., *et al.*: Temporal patterning of neurofilament light as a blood-based biomarker for stroke: a systematic review and meta-analysis. Frontiers in Neurology **13**, 841898 (2022)

[8] Xu, Q., Xu, H., Gu, H., Li, J., Wang, Y., Wei, M.: Development of lateral flow immunoassay system based on superparamagnetic nanobeads as labels for rapid quantitative detection of cardiac troponin I. Materials Science and Engineering: C **29**(3), 702–707 (2009)
