# Supplementary material for: DNA origami signal amplification in lateral flow immunoassays
Source: Nat Commun. 2025 Apr 4;16:3216. doi: 10.1038/s41467-025-57385-6 (PMC11971289; doi:10.1038/s41467-025-57385-6)
Supplement: Supplementary file 2 — Reporting Summary [file 41467_2025_57385_MOESM2_ESM.pdf]

nature portfolio

Corresponding author(s): Heini Ijäs, Maximilian J. Urban

Last updated by author(s): Feb 7, 2025

Reporting Summary

Nature Portfolio wishes to improve the reproducibility of the work that we publish. This form provides structure for consistency and transparency in reporting. For further information on Nature Portfolio policies, see our Editorial Policies and the Editorial Policy Checklist.

Statistics

For all statistical analyses, confirm that the following items are present in the figure legend, table legend, main text, or Methods section.

n/a	Confirmed	
[ ]	[x]	The exact sample size (*n*) for each experimental group/condition, given as a discrete number and unit of measurement
[ ]	[x]	A statement on whether measurements were taken from distinct samples or whether the same sample was measured repeatedly
[x]	[ ]	The statistical test(s) used AND whether they are one- or two-sided<br>*Only common tests should be described solely by name; describe more complex techniques in the Methods section.*
[x]	[ ]	A description of all covariates tested
[x]	[ ]	A description of any assumptions or corrections, such as tests of normality and adjustment for multiple comparisons
[ ]	[x]	A full description of the statistical parameters including central tendency (e.g. means) or other basic estimates (e.g. regression coefficient) AND variation (e.g. standard deviation) or associated estimates of uncertainty (e.g. confidence intervals)
[x]	[ ]	For null hypothesis testing, the test statistic (e.g. *F*, *t*, *r*) with confidence intervals, effect sizes, degrees of freedom and *P* value noted<br>*Give P values as exact values whenever suitable.*
[x]	[ ]	For Bayesian analysis, information on the choice of priors and Markov chain Monte Carlo settings
[x]	[ ]	For hierarchical and complex designs, identification of the appropriate level for tests and full reporting of outcomes
[x]	[ ]	Estimates of effect sizes (e.g. Cohen's *d*, Pearson's *r*), indicating how they were calculated

Our web collection on statistics for biologists contains articles on many of the points above.

Software and code

Policy information about availability of computer code

Data collection	Image Lab software (Bio-Rad Laboratories, Inc., Version 6.0.0 build 25) was used for collecting fluorescence strip and gel images.
Data analysis	Lateral flow strip image analysis was done using ImageJ 1.54f (distribution Fiji 2.15.1). OriginPro 2023 version 10.0.0.154 was used for processing and fitting the data.

For manuscripts utilizing custom algorithms or software that are central to the research but not yet described in published literature, software must be made available to editors and reviewers. We strongly encourage code deposition in a community repository (e.g. GitHub). See the Nature Portfolio guidelines for submitting code & software for further information.

Data

Policy information about availability of data

All manuscripts must include a data availability statement. This statement should provide the following information, where applicable:

- Accession codes, unique identifiers, or web links for publicly available datasets
- A description of any restrictions on data availability
- For clinical datasets or third party data, please ensure that the statement adheres to our policy

The data supporting the findings of this study are available within this article and its Supplementary Information, and from corresponding author(s) upon request. The data generated in this study have been deposited in the Dryad database as a Source Data file under accession code https://doi.org/10.5061/dryad.k6djh9whk

Research involving human participants, their data, or biological material

Policy information about studies with human participants or human data. See also policy information about sex, gender (identity/presentation), and sexual orientation and race, ethnicity and racism.

Reporting on sex and gender	n/a
Reporting on race, ethnicity, or other socially relevant groupings	n/a
Population characteristics	n/a
Recruitment	n/a
Ethics oversight	n/a

Note that full information on the approval of the study protocol must also be provided in the manuscript.

Field-specific reporting

Please select the one below that is the best fit for your research. If you are not sure, read the appropriate sections before making your selection.

☒ Life sciences ☐ Behavioural & social sciences ☐ Ecological, evolutionary & environmental sciences

For a reference copy of the document with all sections, see nature.com/documents/nr-reporting-summary-flat.pdf

Life sciences study design

All studies must disclose on these points even when the disclosure is negative.

Sample size	No statistical method was used to predetermine sample size (not applicable to the presented experiments).
Data exclusions	No data were excluded from the analyses.
Replication	All measurements were reproducible as explained in the manuscript and the supplementary information.
Randomization	The experiments were not randomized (not applicable to the presented experiments).
Blinding	The investigators were not blinded to allocation during experiments and outcome assessment.

Reporting for specific materials, systems and methods

We require information from authors about some types of materials, experimental systems and methods used in many studies. Here, indicate whether each material, system or method listed is relevant to your study. If you are not sure if a list item applies to your research, read the appropriate section before selecting a response.

Materials & experimental systems

n/a	Involved in the study	
☐	☒	Antibodies
☒	☐	Eukaryotic cell lines
☒	☐	Palaeontology and archaeology
☒	☐	Animals and other organisms
☒	☐	Clinical data
☒	☐	Dual use research of concern
☒	☐	Plants

Methods

n/a	Involved in the study	
☒	☐	ChIP-seq
☒	☐	Flow cytometry
☒	☐	MRI-based neuroimaging

Antibodies

Antibodies used	anti-cardiac troponin complex MAb 20C6cc (HyTest, cat.# 4TC2, lot: 22/11-TC2-C6cc)<br>anti-cTnI MAb 19C7cc (HyTest Ltd, Cat.# 4T21/4T21cc, lot: 22/03-T21cc-C7cc)<br>anti-cTnI MAb Y302 (HyTest Ltd., Cat.# RC4T21, lot. 23/08-RC4T21-Y302)<br>anti-NF-L mAb 47:3 (UD1) (Uman Diagnostics, Article No. 27016, Lot. 90188)<br>biotin labelled anti-NF-L mAb 2:1 (UD3) (Uman Diagnostics, Article No. 27018, Lot. 90189)

Validation	Troponin antibodies: all the applied monoclonal antibodies are sold for development of cardiac troponin I sandwich immunoassays; they have been validated and their epitope specificity to human cardiac troponin I (or IC complex) has been determined by the manufacturer (HyTest Ltd.), information available at HyTest website (hytest.fi).<br>NfL antibodies: UD1 and UD3 recognize the conserved rod domain of the neurofilament light protein (NF-L). No cross-reactivity against NF-M, NF-H and GFAP. Validated by the manufacturer, information available at www.umandiagnostics.com

Plants

Seed stocks	n/a
Novel plant genotypes	n/a
Authentication	n/a
